# *In vitro* Effect of Geranylgeraniol (GGOH) on Bisphosphonate-Induced Cytotoxicity of Oral Mucosa Cells

**DOI:** 10.3389/froh.2022.892615

**Published:** 2022-06-20

**Authors:** Krit Rattanawonsakul, George Bullock, Robert Bolt, Frederik Claeyssens, Simon Atkins, Vanessa Hearnden

**Affiliations:** ^1^Department of Materials Science and Engineering, The University of Sheffield, Sheffield, United Kingdom; ^2^School of Clinical Dentistry, The University of Sheffield, Sheffield, United Kingdom

**Keywords:** MRONJ, Geranylgeraniol, fibroblasts, keratinocytes, pamidronic acid, zoledronic acid

## Abstract

Medication-related osteonecrosis of the jaw (MRONJ) is an often-severe complication found in patients receiving bisphosphonates in the management of Paget's, osteoporosis and metastatic bone cancer. Mucosal breakdown with bone exposure is a primary clinical presentation of MRONJ linked to the inhibitory effect of nitrogen-containing bisphosphonates (N-BP) on the mevalonate pathway. Geranylgeraniol (GGOH) has demonstrated a rescue effect on N-BP-treated osteoclasts but the biological effects on oral soft tissues and cells remain unclear. This study aimed to determine whether GGOH could prevent bisphosphonate induced toxicity to oral mucosa cells *in vitro*. Primary oral fibroblasts and keratinocytes were exposed to different GGOH concentrations or GGOH in combination with two nitrogen-containing bisphosphonates, zoledronic acid (ZA) or pamidronic acid (PA), for 72 h. The metabolic activity of each cell type was measured using the 3-(4,5-dimethylthiazol-2-yl)-2,5-diphenyltetrazolium bromide (MTT) assay. GGOH without bisphosphonates significantly reduced the metabolic activity of oral mucosa cells. Fibroblasts treated with GGOH and ZA in combination showed a slight increase in metabolic status compared to fibroblasts treated with ZA alone, however this positive effect was not observed in keratinocytes. In the presence of PA, GGOH was unable to increase the metabolic activity of either cell type. These findings demonstrate that GGOH is toxic to oral mucosa cells and that GGOH was not able to prevent bisphosphonate induced toxicity. These data show that GGOH does not have therapeutic potential for bisphosphonate-induced soft tissue toxicity in MRONJ and the use of GGOH as an MRONJ treatment should be strongly reconsidered.

## Introduction

Medication-related osteonecrosis of the jaw (MRONJ) is an adverse event caused by antiresorptive and antiangiogenic drugs, and is characterized by exposed, necrotic bone without mucosal healing after 8 weeks [1, 2]. The disease predominantly occurs in patients receiving intravenous nitrogen-containing bisphosphonates (N-BPs) such ZA or PA for the treatment of bone malignancies [3]. MRONJ can cause significant morbidity in terms of pain, discomfort, and dysfunctional oral habits which worsen the quality of life [4, 5].

Though the disease was first identified almost 20 years ago [6], the definitive pathophysiology of MRONJ has not yet been defined and is likely multifactorial [7]. Multiple contributing mechanisms have been proposed since the disease process was first characterized; including bone turnover impairment, angiogenesis inhibition, infection and inflammation, and mucosal toxicity [4, 7]. A loss of mucosal covering leading to the exposure of bone is a clinical hallmark of MRONJ and an important target in the development of novel therapies [8]. Previous studies have demonstrated clinically relevant concentrations of both ZA and PA can induce significant toxicity in the cells and *in vitro* tissues of the oral mucosa [9–11] and that this interferes with the oral wound healing process [3, 12], highlighting the significance of soft tissue toxicity in the development and resolution of MRONJ.

The clinical management of MRONJ is challenging as there is limited data on its pathogenesis and there has recently been controversy over the current therapeutic strategies [7, 13]. The key factors in MRONJ management include (i) necrotic bone removal, (ii) soft tissue restoration, and (iii) pain and infection control [14]. Currently, there is no standard treatment protocol for MRONJ [15]. Research is now required to develop alternative therapeutic measures to help manage the disease more effectively.

**Figure 1:**
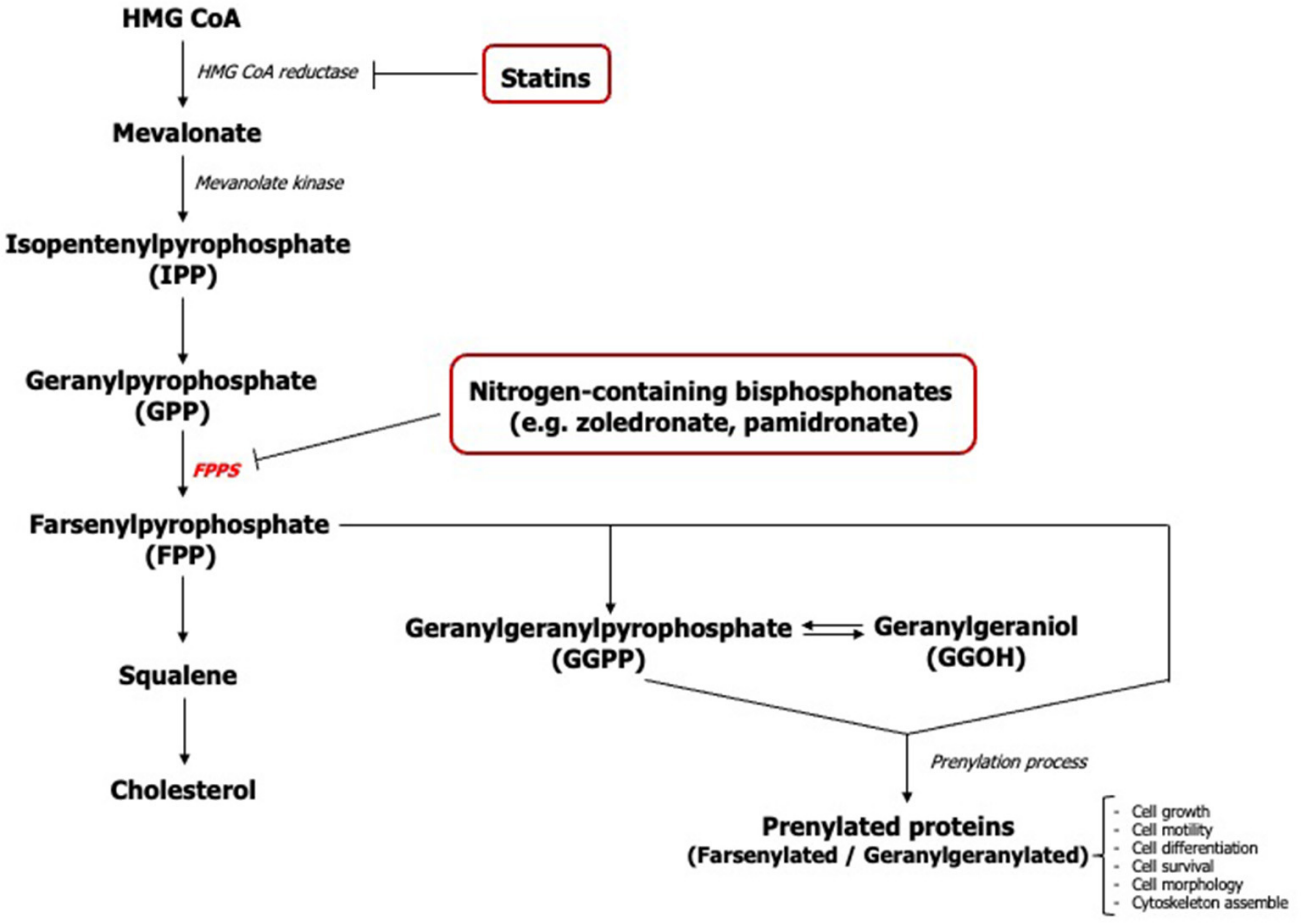
The mevalonate pathway - FPPS (Farsenylpyrophosphate synthase).

The action of nitrogen-containing bisphosphonates primarily inhibits the farsenylpyrophosphate synthase (FPPS) enzyme of the mevalonate pathway causing disruption in the synthesis of isoprenoids including farsenyl pyrophosphate (FPP) and geranylgeranyl pyrophosphate (GGPP) (Figure 1) [16]. The loss of these mevalonate intermediates negatively affects the prenylation of GTP-binding proteins such as Ras, Rho, Rac, Rap, and Cdc42 which are necessary for the growth, differentiation, and function of osteoclasts [17, 18] along with other cell types.

Geranylgeraniol (GGOH), an analog molecule of GGPP, has previously been shown to play a pivotal role in the viability and proliferation of mesenchymal stem cells [19]. It has been demonstrated that GGOH counteracted bisphosphonate toxicity in several cell types including osteoclasts, osteoblasts, endothelial cells, keratinocytes, and fibroblasts [2, 3, 16, 20, 21]. However, previous studies of GGOH on cells of the oral mucosa have shown inconsistent and contradictory findings. Most studies were undertaken using a single GGOH concentration ranging between 0.5 to 50 μM to reverse the effect of bisphosphonates [1, 3, 16, 20, 22, 23] and very few studies have reported the cytotoxic effect of GGOH when exposed to cells in the absence of bisphosphonates [1]. Therefore, further studies are needed to define the *in vitro* function of GGOH on the oral mucosa and to determine an effective dose.

GGOH has also been tested in *in vivo* studies. MRONJ-induced rats exposed to GGOH demonstrated an improvement in oral wound healing [17]. Inflammatory tissues with favorable signs of tissue remodeling were observed in rats receiving 5 mM GGOH once daily in combination with ZA, when compared to a control group solely treated with ZA, suggesting the potential positive effect of GGOH on the healing of MRONJ wounds.

GGOH has not only attracted interest in the potential management of MRONJ, but also in further clinical applications in the management of cancer and drug complications as a result of its anti-inflammatory, antibacterial [24, 25] and anti-cancer properties [26–28]. GGOH has been found to be capable of inducing cellular apoptosis and reducing the viability of various cancer cells including: hepatoma, prostate carcinoma, or colon cancer cells [26–28]. Other studies have shown that myotoxicity, the most common side effect of statins, can be prevented with GGOH [29].

The aim of this study was to analyse the effect of GGOH on oral mucosa cells in both the presence and absence of clinically-relevant bisphosphonates, so as to determine the molecule's potential as a treatment for soft tissue damage in MRONJ. We hypothesized that GGOH could restore soft tissue impairment in MRONJ by supplementing the depletion of geranylated proteins caused by bisphosphonates in cells of the oral mucosa.

## Materials and Methods

### Cell Culture

Three cell types were used for the experiments. Human immortalized oral keratinocytes containing telomerase reverse transcriptase (OKF6/TERT-2) [30] were cultured in keratinocyte serum free medium (KSFM) supplemented with 0.05 mg/ml bovine pituitary extract, 0.005 μg/ml human recombinant epidermal growth factor (all from Gibco, Paisley, UK), and penicillin/streptomycin 100 μg/ml (Sigma-Aldrich, Dorset, UK). Human primary normal oral fibroblasts (NOFs) and keratinocytes (NOKs) were isolated from buccal biopsies as previously described elsewhere [31]. Written informed consent was obtained from volunteers before the collection of buccal biopsies and experimental protocols were ethically approved by the University of Sheffield Research Ethics Committee (Reference number 003463). All procedures were performed in accordance with the Declaration of Helsinki. Primary fibroblasts were grown in Dulbecco's Modified Eagle Medium (DMEM) (Sigma-Aldrich) with 10% fetal calf serum (FCS) (Biosera, East Sussex, UK), 0.01 mg/ml L-glutamine (Sigma-Aldrich), and 100 μg/ml penicillin/streptomycin (Sigma-Aldrich). Primary keratinocytes were cultured on a feeder layer of irradiated mural fibroblasts (i3T3) in Green's medium made from 3:1 mixture of DMEM and Ham's Nutrient Mixture F12 supplemented with 10% FCS (Biosera), 0.01 mg/ml L-glutamine, 100 μg/ml Penicillin/Streptomycin, 0.625 μg/ml Fungizone, 0.025 μg/ml adenine, 1.36 ng/ml/ 5 μg/ml of 3,3,5-Tri-iodothyronine /Apo-Transferrin (T/T), epidermal growth factor 5 ng/ml, Insulin 5 μg/ml, hydrocortisone 4 μg/ml, and cholera toxin 8.47 ng/ml (all from Sigma-Aldrich except FCS). All cell types were grown in humidified conditions in a 5% CO_2_ incubator at 37°C.

### Geranylgeraniol (GGOH) and Bisphosphonates

A stock solution of GGOH (20 mM) (Sigma-Aldrich) was prepared in ethanol. It was aliquoted and stored at −20°C. The solution was thawed and diluted with cell culture medium before each experiment. The working concentration of GGOH used in this study was from 0.5 to 100 μM (0.5, 1, 2.5, 5, 10, 25, 50 and 100 μM). Two nitrogen-containing bisphosphonates, pamidronate disodium salt anhydrate (PA) and zoledronic acid monohydrate (ZA) (Sigma-Aldrich), were used in this study. The concentrations of 100 μM PA and 10 μM ZA were chosen based on previously published work [31]. The maximum concentration of the ethanol vehicle did not exceed 0.5% (v/v) which did not cause significant toxicity in any of the cell types tested (data not shown).

### Cell Viability

Cells were seeded in culture plates at an optimum density (NOF: 10,000 cells/cm^2^), (OKF6/TERT-2: 16,700 cells/cm^2^), (NOK: 10,000 cells/cm^2^ with i3T3: 5,000 /cm^2^) and left to adhere for 24 hours. The following day, the medium was replaced with fresh medium containing different concentrations of GGOH or GGOH in combination with either 10 μM ZA or 100 μM PA. The viability was measured every 24 h for 3 days.

Cellular metabolic activity was measured using the 3-(4,5-dimethylthiazol-2-yl)-2,5-diphenyltetrazolium bromide (MTT) (Sigma-Aldrich, Dorset, UK) assay according to the manufacturer's instructions. Metabolically active (viable) cells convert a yellow tetrazolium salt to purple formazan. At each time point, cells were washed once with sterile PBS and incubated with 0.5 mg/ml MTT solution for 90 min. Acidified isopropanol was then added to solubilise the formazan crystals and absorbance was read at 540 nm. Results from each condition were normalized to the absorbance value of untreated cells cultured for 24-h.

### Morphological Evaluation

Cell morphology was examined under a light inverted microscope (Motic AE 2000). Images were captured using a digital camera (Moticam 2) and Motic image 2.0 Plus software.

### Statistical Analysis

Values were presented as mean ± standard deviation (SD). Three independent experiments were conducted (*N* = 3) and technical triplicates were used for each experiment (*n* = 3), unless indicated otherwise. All statistical analyses in this study were performed by using Prism 9 software (GraphPad, San Diego, CA, USA). The difference between each group was determined using a two-way analysis of variance (two-way ANOVA). Dunnett's *post-hoc* test was used to compare between the experimental and control groups at each time point. Statistical significance was considered when the *p*-value was below 0.05.

## Results

### GGOH Cytotoxicity on Oral Mucosa Cells

**Figure 2:**
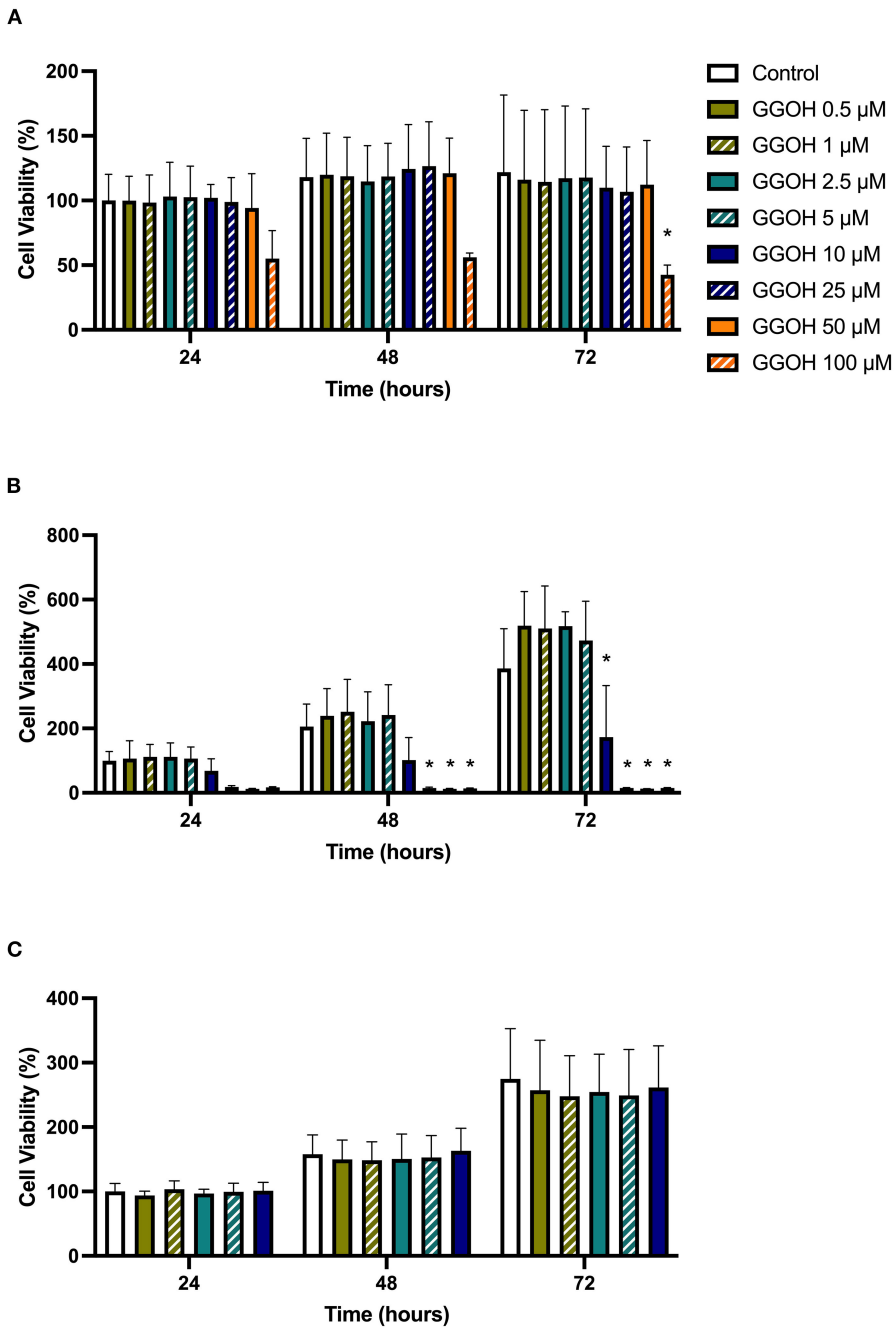
The metabolic activities of **(A)** NOFs **(B)** OKF6/TERT-2 **(C)** NOKs after treated with different GGOH concentrations over 72 h of treatment. All *N* = 3 except control and GGOH 10 μM of **(B)**, *N* = 6. Mean +/- standard deviation (SD). Significance (*) was indicated when *p ≤ 0.05 in comparison to GGOH 0 μM.

**Figure 3:**
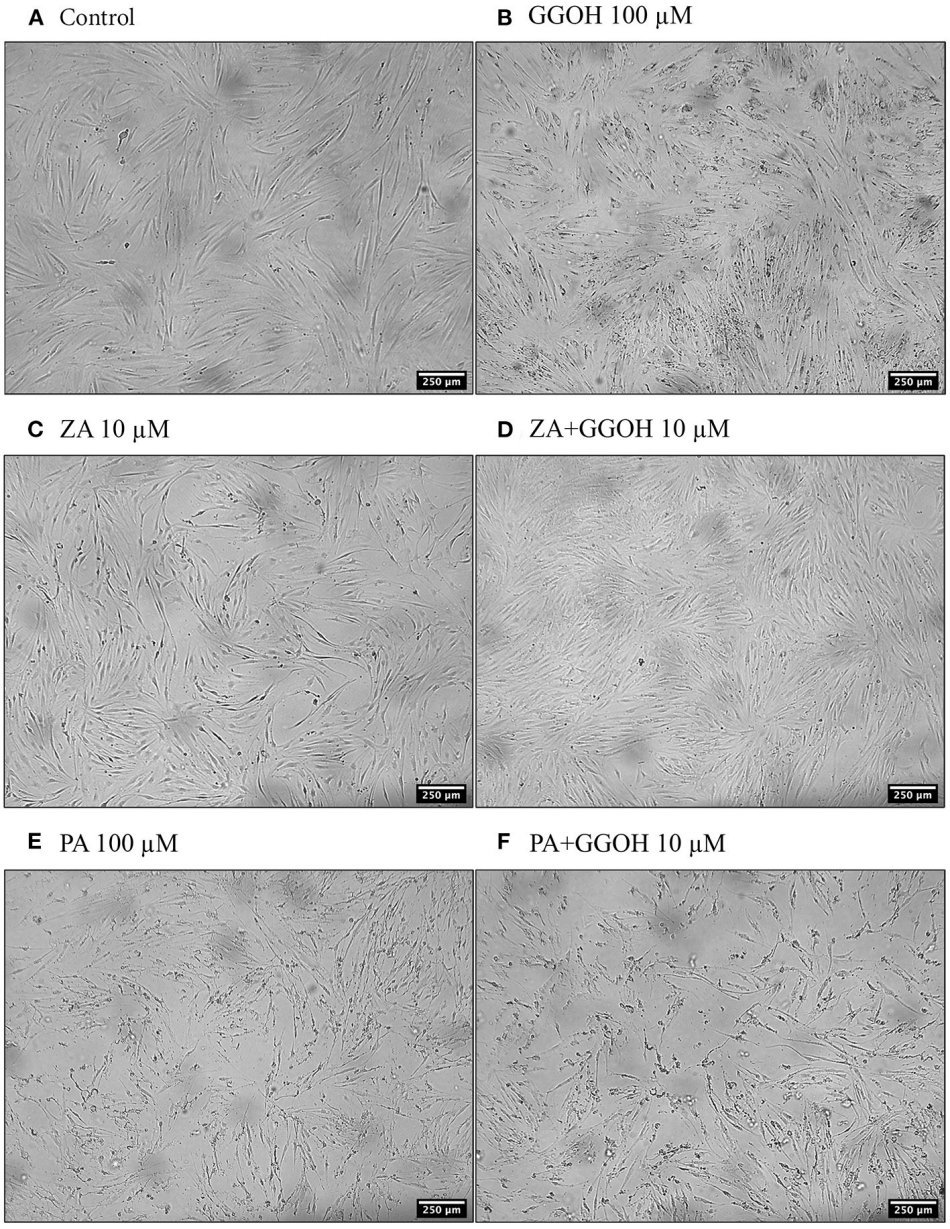
The morphology of oral fibroblasts after 72 h of treatment **(A)** Control **(B)** GGOH 100 μM **(C)** ZA 10 μM **(D)** ZA+GGOH 10 μM **(E)** PA 100 μM **(F)** PA+GGOH 10 μM. Magnification of 4× from a light inverted microscope.

The metabolic activities of oral mucosa cells in response to different GGOH concentrations after 72 h were measured using the MTT assay, and the results are illustrated in Figure 2. There were no changes in viability when fibroblasts were cultured with low GGOH doses (0.5–50 μM) while the highest GGOH concentration (100 μM) reduced the viability over the experimental period. The toxicity from 100 μM GGOH on fibroblasts was only statistically significant following 72-h exposure (*p* < 0.05) (Figure 2A), which is consistent with the changes to fibroblast morphology, as shown in Figure 3B.

**Figure 4:**
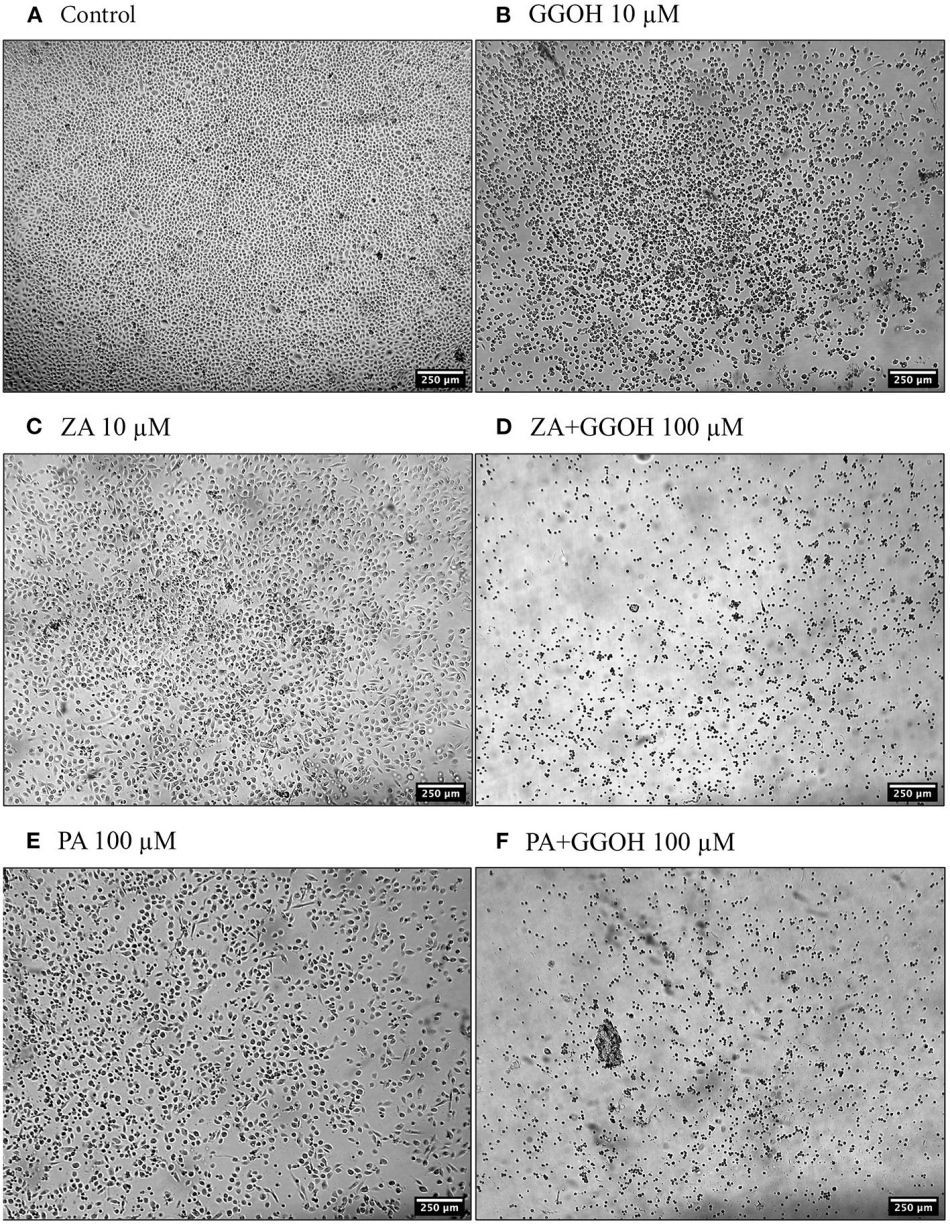
The morphology of oral keratinocytes after 72 h of treatment **(A)** Control **(B)** GGOH 10 μM **(C)** ZA 10 μM **(D)** ZA+GGOH 100 μM **(E)** PA 100 μM **(F)** PA+GGOH 100 μM. Magnification of 4× from a light inverted microscope.

The treatment of immortalized keratinocytes with 0.5–5 μM GGOH did not affect the metabolic activity at any time point. However, concentrations of 25 μM and above of GGOH markedly reduced the cellular viability after 48 hours (*p* < 0.05). At 72 h, 10 μM GGOH significantly reduced OKF6 viability (*p* < 0.05) (Figure 2B). A microscopic image (Figure 4B) demonstrated the unattached rounded cells, indicating dead cells.

Only GGOH doses from 0.5 to 10 μM were used to examine the effect of GGOH on primary oral keratinocytes (NOKs) because of the observed toxicity in OKF6. Figure 2C demonstrates the cellular viability of NOKs after incubation with GGOH for 72 h. There were no significant changes in the viability from all GGOH concentrations at any time points.

### GGOH Effect on ZA-Induced Toxicity of Oral Mucosa Cells

**Figure 5:**
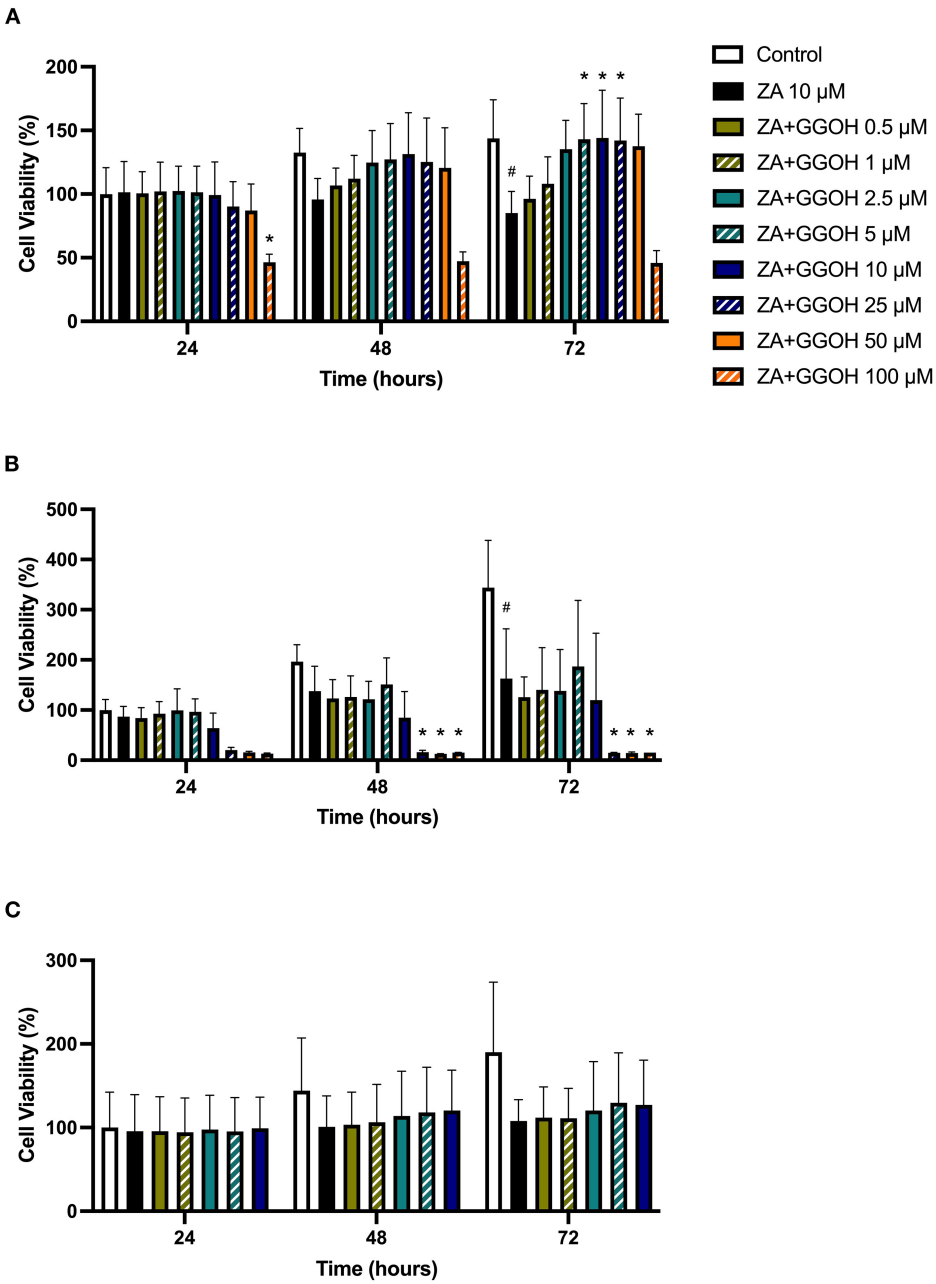
**(A)** NOF **(B)** OKF6/TERT-2 **(C)** NOK viability in the presence of different GGOH concentrations in combination with ZA for 72 h. All N = 3 except control, ZA, and ZA+GGOH 10 μM of **(B)**, N = 6. Mean +/− standard deviation (SD). # shows statistical significance (p < 0.05) in comparison to untreated control and * shows statistical significance (p < 0.05) between 10 μM ZA group and test condition.

To determine the ability of GGOH to reverse the toxicity of bisphosphonates, the cellular viability of oral mucosa cells in the presence of 10 μM ZA with different GGOH doses was assessed. When fibroblasts were incubated with ZA-containing media without GGOH, the alteration of cell morphology was detected under the microscope (Figure 3C). The metabolic activities were negatively affected and significant toxicity was observed at the 72-h time point (*p* < 0.05). The combination treatment of 100 μM GGOH and 10 μM ZA caused a significant reduction of metabolic activities after 24 h, indicating GGOH toxicity. At 48 and 72 h, the addition of GGOH doses from 0.5 to 25 μM was able to increase the viability of ZA-treated fibroblasts in a dose-dependent manner compared to fibroblasts treated with ZA without GGOH. Three GGOH doses (5, 10, and 25 μM) significantly increased the metabolic activity of cells compared to control levels after 72 h (*p* < 0.05) (Figure 5A). The increased confluence of fibroblasts in the presence of ZA and GGOH 10 μM is shown in Figure 3D.

Figure 5B demonstrates that 10 μM ZA was toxic to immortalized keratinocytes with a significant reduction in the viability after 72 h, which is correlated with the morphological changes shown in Figure 4C. GGOH did not increase the viability of ZA treated OKF6/TERT-2 at any time point or at any concentration tested. Instead, the combination of GGOH treatment (25 μM and above) with 10 μM ZA led to significantly lower metabolic activities in OKF6/TERT-2 cells treated in combination (*p* < 0.05). The morphological analysis in Figure 4D illustrates floating cells and cellular debris, confirming the toxic effect of ZA and GGOH on keratinocytes.

The toxicity of ZA was also observed in NOKs following culture with 10 μM zoledronate for 72 h; however a statistical significance was not found, as shown in Figure 5C. Treatment with GGOH (0.5 to 10 μM) had no effect on the metabolic activity of cells.

### GGOH Effect on PA-Induced Toxicity of Oral Mucosa Cells

**Figure 6:**
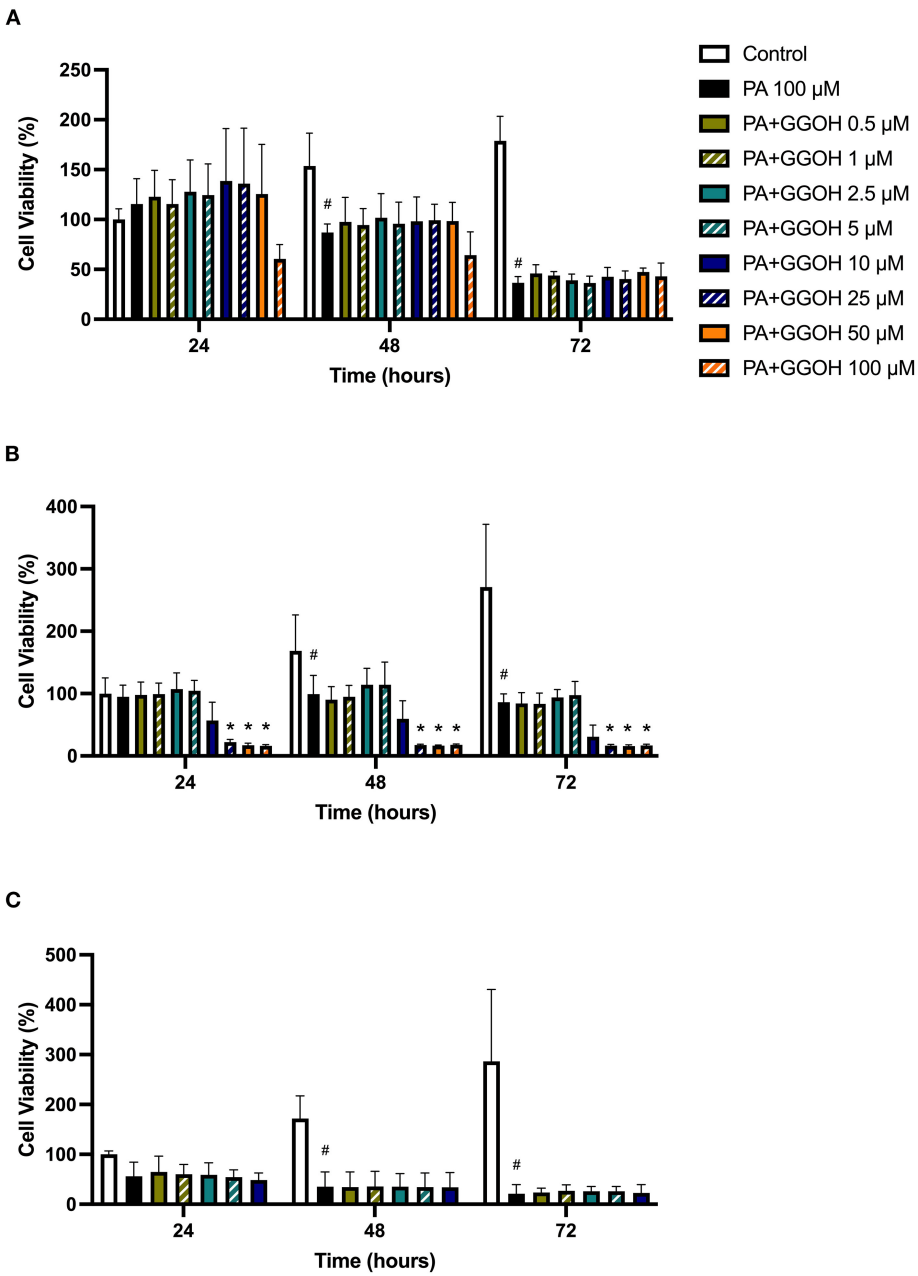
**(A)** NOF **(B)** OKF6/TERT-2 **(C)** NOK viability in the presence of different GGOH concentrations in combination with PA for 72 h. All **(A,B)** N = 3 except Control, PA, and PA+GGOH 10 μM of **(B)** N = 4, all conditions of **(C)** N = 2. Mean +/− standard deviation (SD). # shows statistical significance (p < 0.05) in comparison to untreated control and * shows statistical significance (p < 0.05) between 100 μM PA group and test condition.

Since PA has lower potency than ZA, a higher dose of PA (100 μM) was used to induce the toxicity on oral mucosa cells. Figure 6A illustrates the response of PA-treated fibroblasts to different GGOH concentrations over 72 h. PA produced a significant toxic effect at 48, and 72 h, as shown by a reduction the metabolic activity to approximately 90, and 40%, respectively (*p* < 0.05). There were no differences in the metabolic activities from GGOH plus PA conditions at any time points, indicating that GGOH had no protective effect on PA-induced toxicity in oral fibroblasts. The alteration of fibroblast structure and morphology in the presence of PA, and PA with GGOH was also presented in Figures 3E,F, clearly demonstrating the toxicity.

When OKF6/TERT-2 cells were treated with PA, the viability was significantly decreased after 48 and 72 h. The addition of different GGOH doses again had no rescue effect on the viability of immortalized keratinocytes in the presence of PA. Instead, the addition of 25 μM GGOH and above negatively affected the metabolic activities of OKF6/TERT-2 at all time points (*p* < 0.05) (Figure 6B). Morphological changes of keratinocytes were seen in Figures 4E,F.

Figure 6C shows the metabolic activities of NOKs following incubation with PA and GGOH. PA alone reduced the viability at all time points. No rescue effect of GGOH was observed from any concentrations on PA-treated cells.

## Discussion

The absence of effective treatment options has driven efforts to develop novel therapies for patients affected with MRONJ. Non-healing mucosal wounds resulting in the exposure of necrotic bone are the primary feature of MRONJ and are responsible for many of the symptoms including loss of function, infection and pain [8]. Therefore, the restoration of the soft tissue barrier is expected to support resolution of the disease and GGOH has been identified as a molecule of interest in MRONJ [16, 20].

GGOH, an isoprenoid molecule that can be converted to GGPP in the mevalonate pathway, has been proposed as a potential tool to overcome bisphosphonate toxicity by salvaging the loss of geranylated molecules to maintain normal cellular activities. GGOH's other biological activities, including its restorative effects in N-BP treated osteoclasts [2] and its anti-inflammatory and antimicrobial features [24, 25], led to our hypothesis that GGOH could improve mucosal integrity and wound healing in patients treated with N-BPs.

In this study, we evaluated the *in vitro* effects of GGOH on oral fibroblasts and keratinocytes, the cells responsible for oral wound healing and mucosal integrity [32], in combination with N-BPs to investigate the potential of GGOH to reduce soft tissue toxicity.

Prior to the evaluation of GGOH therapeutic effects on bisphosphonate-induced soft tissue toxicity, the cytotoxic study of GGOH alone was necessary to verify its safety profile. We have demonstrated that low GGOH doses had no effect on the viability of keratinocytes and fibroblasts, in line with previous studies showing GGOH concentrations between 0.5 to 10 μM produced minimal toxicity to oral mucosa cells [20–22]. However, the metabolic activity of cells was negatively affected with GGOH doses of 10 μM and above in OKF6/TERT-2. To the best of our knowledge, the response of oral keratinocytes to this range of GGOH concentrations has not been reported before. This is the first study demonstrating the toxic effect of GGOH on oral keratinocytes which is important when considering GGOH based therapies for mucosal healing. Following these results, experiments using higher GGOH doses on primary keratinocytes were suspended.

Fibroblasts were less susceptible to GGOH toxicity than keratinocytes as the tolerated dose was higher (50 μM vs. 5 μM). Our findings on fibroblasts are consistent with previous studies showing that 50 μM GGOH did not cause any adverse effect on fibroblast viability [3, 16]. However, the toxicity of GGOH at a similar concentration has also been reported [1]. Zafar et al. demonstrated a significant reduction in the viability of gingival fibroblasts after treatment with a single dose of 50 μM GGOH [1]. It is worth noting that earlier studies did not fully evaluate the responses of oral mucosa cells to GGOH exposure, studying only a single GGOH concentration to examine the beneficial role of GGOH on counteracting bisphosphonate toxicity. Here, we provide results on the impact of different GGOH doses on oral mucosa cell toxicity which presents a more complete picture of the dose dependent effects of GGOH.

Though unwanted toxicity from individual GGOH treatment was found, the key aim of this study was to determine whether GGOH can protect cells from bisphosphonate induced toxicity. We used two nitrogen-containing bisphosphonates, ZA and PA, in this study since they are most associated with the risk of developing MRONJ [3]. The selected concentrations for both ZA and PA were clinically relevant and previously reported to be toxic to oral mucosa cells [3, 31]. In the present study, cells were cultured with different GGOH concentrations in combination with either ZA or PA simultaneously. We have shown that GGOH increased the viability of ZA-treated fibroblasts, but GGOH was unable to increase the viability in PA-treated cells. This was consistent with previous studies that showed GGOH successfully increased cellular viability in ZA treated cells [1, 3, 16, 21]. Interestingly, GGOH was able to restore the metabolic activity of fibroblasts to levels comparable with the untreated control group, indicating a cytoprotective effect in ZA treated cells. In terms of PA, the effects of GGOH were different between each study. Our findings support the work by Ziebart et al. that showed GGOH had no effect on cell viability where even lower PA concentrations at 5 or 50 μM were used [21]. On the contrary, Cozin et al. demonstrated that GGOH increased the metabolic activity of gingival fibroblasts if incubated with 30 μM PA, but saw no positive effect from 60 μM PA treatment [3], suggesting PA concentration influences the success of GGOH in preventing toxicity.

Meanwhile, GGOH failed to restore the metabolic activities of immortalized and primary oral keratinocytes from bisphosphonate toxicity and high doses of GGOH worsened the cellular viability of OKF6/TERT-2. Our findings are distinct from recent studies that reported the therapeutic effect of GGOH in keratinocytes. Kim et al. demonstrated that 0.5 μM GGOH worked effectively against PA induced toxicity, while Pabst et al. showed 10 μM GGOH had a positive effect on primary keratinocyte viability [20, 22]. Our results also showed that the combination treatment of PA and GGOH appear to be more toxic to primary keratinocytes compared to the immortalized cell line.

The different responses observed in fibroblasts and keratinocytes may be related to differences in mitochondrial activity between these cells types and their response to GGOH. Keratinocytes appear to be more sensitive to the toxicity of GGOH and bisphosphonates than fibroblasts, however, further exploration is required to confirm the mechanism. The observed differences between data shown here and those reported in the literature may be related to the variability in cell sources (particularly for primary cells isolated from different location of oral tissues such as gingiva, buccal mucosa or floor of mouth, and different patients where the variability are well known), incubation time, and evaluation methods.

The MTT assay, used in this study measured mitochondrial metabolic activity of cells as an indirect measure of cell viability. As with all viability assays there are limitations in this technique [33], however, the MTT assay is currently used as the gold standard assay to measure cytotoxicity [33] and has been previously used in GGOH studies [3, 16, 20, 22].

Based on our findings, GGOH appears to have a very narrow therapeutic window that makes it unsuitable for clinical use. The lowest dose of GGOH able to restore fibroblast viability in the presence of ZA was 5 μM however this same dose was unable to preserve the viability of keratinocytes and a small increase in dose (10 μM of GGOH) produced significant toxicity which could lead to further mucosal breakdown or other unwanted off-target effects.

Increasing GGOH levels could also produce a negative consequence in myeloma patients, which form a significant proportion of those suffering with MRONJ. A previous study has indicated that the loss of GGPP impaired the proliferative capacity of myeloma cells [34]. Thus, the addition of GGOH could have the potential to stimulate the proliferation of cancer cells and worsens the disease, precluding the use of GGOH in patients with malignancies or at risk of malignancy.

Taken together, there are a few possible explanations for the failure of GGOH to protect oral soft tissues from bisphosphonate treatment. Here we have shown that GGOH itself impairs the metabolic activity and therefore viability of oral mucosa cells and in some cases this impairment is compounded by the addition of bisphosphonates suggesting a synergistic effect in these cells. An alternative hypothesis is that the cytotoxic effect of bisphosphonates in cells of the oral mucosa may not occur through the mevalonate pathway (as is the case in osteoclasts); meaning GGOH is unable reverse the toxicity induced via this route to protect oral mucosa cells.

Although we have demonstrated that a narrow range of GGOH concentrations can reduce the toxicity caused by ZA in oral fibroblasts, the same restorative effect was not observed in keratinocytes. Marginally higher GGOH doses were shown to cause significant toxicity in oral keratinocytes and the combination of GGOH and N-BPs were in some cases synergistic. Therefore, the use of GGOH to treat bisphosphonate-induced soft tissue damage in MRONJ is not supported by the data presented here and its use in other applications should be carefully considered.

## Data Availability Statement

The original contributions presented in the study are included in the article/supplementary material, further inquiries can be directed to the corresponding author/s.

## Ethics Statement

The studies involving human participants were reviewed and approved by the University of Sheffield Research Ethics Committee. The patients/participants provided their written informed consent to participate in this study.

## Author Contributions

All authors contributed to the study conception and design. Material preparation and data collection and analysis were performed by KR, GB, and VH. The first draft of the manuscript was written by KR. All authors commented on previous versions of the manuscript. All authors contributed to the article and approved the submitted version.

## Funding

This research was supported by a scholarship from the Faculty of Dentistry, Mahidol University, Thailand awarded to KR, and a DTA studentship awarded to GB from the Engineering and Physical Sciences Research Council (EPSRC) through the University of Sheffield. The work was also supported by an EPSRC Doctoral Prize Fellowship awarded to GB (grant code: X/013296), and an EPSRC Impact Acceleration Account grant (grant code: X/167000).

## Conflict of Interest

The authors declare that the research was conducted in the absence of any commercial or financial relationships that could be construed as a potential conflict of interest.

## Publisher's Note

All claims expressed in this article are solely those of the authors and do not necessarily represent those of their affiliated organizations, or those of the publisher, the editors and the reviewers. Any product that may be evaluated in this article, or claim that may be made by its manufacturer, is not guaranteed or endorsed by the publisher.
